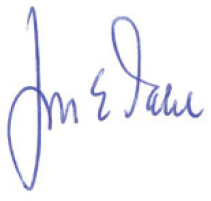# Recipient of Biomaterial Investigations in Dentistry’s Young Author Award 2023

**DOI:** 10.2340/biid.v11.41367

**Published:** 2024-08-22

**Authors:** Anne Peutzfeldt, Jon E. Dahl

**Figure:**
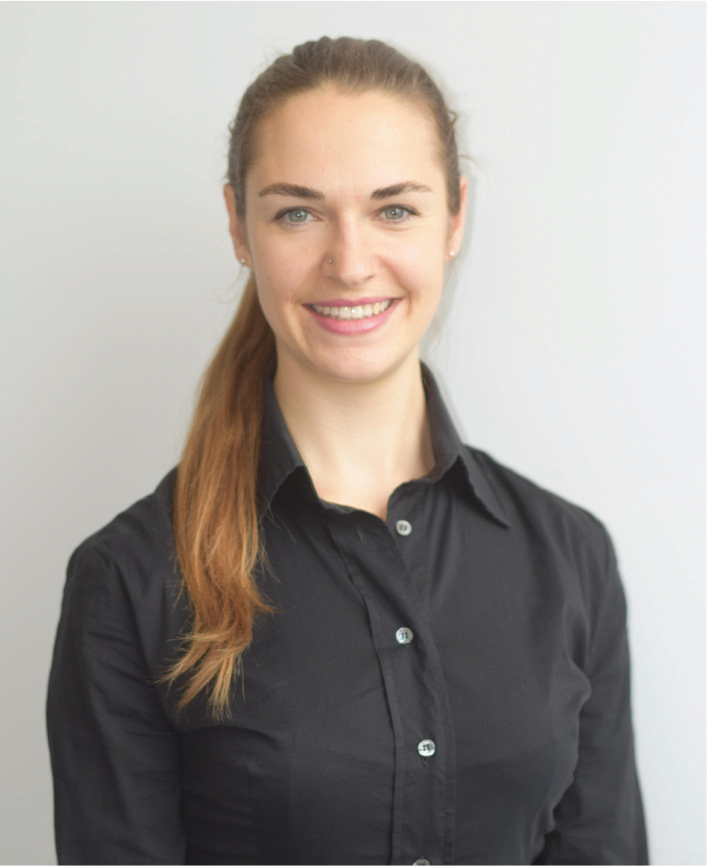


Dear Reader,

We are delighted to announce the recipient of the 2023 Young Author Award: Dr. Clara Isabel Anton y Otero from the Division of Cariology and Endodontology, University Clinics of Dental Medicine, Faculty of Medicine, University of Geneva, Switzerland. Dr Anton y Otero receives the award for her paper: ‘Evaluating the use of self-conditioning adhesive combined with dual curing resin cement as an endodontic sealer: An in vitro study’. The paper was published online on November 18, 2023, and co-authored by Drs. Nicolas Liaudet, Enrico di Bella, Marwa Abdelaziz, Albert Feilzer, Ivo Krejci and Laurine Marger.

The nomination was motivated as follows: The paper introduces a novel method that visualizes the marginal seal of endodontic sealers and explains and discusses the methodology in detail. The results are well presented, accompanied by illustrative images, and thoroughly and systematically discussed. The paper is straightforward to read and readily comprehensible.

There were 10 eligible candidates, and the papers were evaluated based on the following criteria: originality of the study, suitability of the study design, presentation of the results, and readability of the paper.

The award is accompanied by a prize of € 5.000 Euro and a diploma.

The award aims to encourage young scientists to publish their research in Biomaterial Investigations in Dentistry and to showcase what is a good manuscript. The award is presented to a first author who at the time of submission of his/her manuscript is within 10 years of completing his/her last terminal degree (PhD, DDS, DMD, MD, etc.).

Our sincere congratulations to Dr. Anton y Otero and her colleagues.


Anne Peutzfeldt Dr.Odont., PH.D., D.D.S. *Editor-in-Chief* 
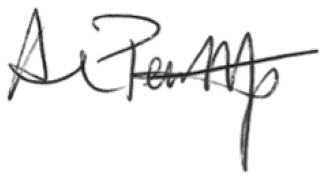

Jon E. Dahl Dr. Odont., Dr. Scient. *Associate Editor*